# Quality of Life in Adult Patients With Epilepsy in Lebanon: A Cross-Sectional Study

**DOI:** 10.7759/cureus.30254

**Published:** 2022-10-13

**Authors:** Layal Abou Zeki, Chawkat El Beaini, Wael Radwan, Taghrid El Hajj

**Affiliations:** 1 Neuroscience Research Center, Lebanese University, Beirut, LBN; 2 Neurology, Ain Wazein Medical Village, Ain Wazein, LBN; 3 Neurology, Aley Specialty Clinic, Aley, LBN; 4 Neurology, Rafic Hariri University Hospital, Beirut, LBN

**Keywords:** seizure, emotional wellbeing, quality of life in epilepsy scale-31, antiepileptic drug treatment, neurological disorder, lebanon, burden, quality of life (qol), epilepsy

## Abstract

Background: Epilepsy is a chronic and heterogeneous neurological disorder that impairs the quality of life (QOL) of sufferers and affects their mental health in many aspects. Few studies have been conducted in the Middle East, especially in Lebanon, on the QOL of patients with epilepsy. The aim of our study is to assess the burden of epilepsy on the life of adult patients in Lebanon and measure the impact of sociodemographic and clinical factors on the QOL.

Materials and Methods: A total of 47 patients from several neurology clinics were included in our study and asked to fill out a questionnaire including the sociodemographic variables and epilepsy-related factors. We used the Quality of Life in Epilepsy Scale-31 (QOLIE-31), which is a 31-question form covering seven attributes of the QOL; overall QOL, emotional wellbeing, social functioning, cognition, energy, seizure worry and medication effects, to assess the QOL of adult patients with epilepsy. Analysis was conducted using SPSS program version 23.

Results: The mean age of our sample was 37.54 years, 53.2% of which were males. The majority were unemployed, educated, and belonging to the medium socioeconomic level. The highest subscale score was for the social functioning subscale with a mean of 74.49±25.89 and the lowest subscale score was for seizure worry with a mean of 55.81±27.14. Employment status, nationality, and socioeconomic level were shown to be associated with the QOL scores. There were no correlations between the clinical factors and the QOL scores.

Conclusion: Sociodemographic factors, namely Lebanese nationality, employment status, and socioeconomic level, were associated with better QOL, reflected by the significant associations between the following parameters and the QOL scores (p-value<0.05), highlighting the positive influence of social support on the outcome of the disease. However, epilepsy-related clinical features did not show any correlation with the QOL and its subscales.

## Introduction

Epilepsy is a heterogeneous and serious neurological disorder that affects the quality of life (QOL) of patients and impacts their social, cognitive, and psychological wellbeing. The International League Against Epilepsy (ILAE) defined epilepsy to be a disease of the brain that is diagnosed under any of the following conditions: (i) at least two unprovoked (or reflex) seizures occurring in more than 24 hours apart, (ii) one unprovoked (or reflex) seizure and a probability of further seizures similar to the general recurrence risk (at least 60%) after two unprovoked seizures occurring over the next 10 years, or (iii) having an epilepsy syndrome [1]. The WHO estimated that there are around 50 million patients with epilepsy in the world, of whom more than 85% live in developing countries. Specifically, the number of people diagnosed with epilepsy who live in the Mediterranean region was estimated to be 4.7 million in 2010 [2]. Other studies estimated people suffering from epilepsy in the Arab world to be 724,500 with a median prevalence of 2.3 per 1000 [3]. Data is still lacking regarding the prevalence of epilepsy in many developing countries, including Lebanon.

People diagnosed with epilepsy usually feel stigmatized and many suffer from depression and anxiety, which can lead to suicidal ideation [4,5]. Several studies have reported that epileptic patients have a poorer QOL as compared to controls [6-8]. Furthermore, several studies have reported that their QOL is influenced by the clinical characteristics of epilepsy (type of seizures, seizure frequency, number of antiepileptic drugs (AEDs), and duration of epilepsy), as well as some sociodemographic variables (age, marital status, employment status) [9-11].

The duration of epilepsy might also play an important role in predicting the QOL of people diagnosed with epilepsy. The term “new-onset epilepsy” is defined to include any patient with evidence of two or more seizures within the last 12 months. It is considered to be a subcategory of “Newly Diagnosed Epilepsy” by several references; the latter is diagnosed if there’s evidence of ongoing seizures for more than one year [12,13]. Herodes et al. reported lower scores reflecting poorer QOL with a shorter duration of epilepsy with significant effects on energy, emotional wellbeing, and bodily pain [14]. 

The primary aim of our study was to assess the QOL of patients suffering from epilepsy in Lebanon. Our secondary aim was to identify different sociodemographic and clinical factors affecting their health-related QOL (HRQOL).

## Materials and methods

A cross-sectional study was conducted after obtaining approval from the Rafic Hariri University Hospital Institutional Review Board, Beirut, Lebanon (approval number: FWA00016332). Participant enrollment was initiated on June 1, 2018, and ended by the end of July 2018. An overall sample of 47 patients, in Mount Lebanon and Beirut, was collected. Patients from three hospitals, one of which is public, and three medical centers were contacted. Written informed consent was obtained from the patients for participation in this study and the publication of the findings.

Patients’ QOL was assessed using the Quality of Life in Epilepsy Scale-31 (QOLIE-31). We used this questionnaire specifically since it is the only questionnaire translated into Arabic and validated. Furthermore, it covers several important aspects regarding the QOL [15]. It is a short questionnaire derived from QOLIE-89 [16] and comprises seven multisystem scales that assess several health domains: emotional wellbeing, energy/fatigue, cognition, seizure worry, social functioning, medication effects, and overall quality of life. The total QOL scores were then calculated using the QOLIE-31 scoring manual. Scores range on a scale from 0-100 where higher scores reflect better quality of life [17].

Patients aged 18 years and above and diagnosed with epilepsy by a neurologist were included in the study. Patients aged less than 18 years or suffering from mental retardation, cognitive disorders, and neurodegenerative and psychiatric diseases were excluded from the study for two reasons. First, the questionnaire is a self-reported one requiring the patient to mentally be able to answer the questions. Second, these disorders could affect the QOL, independent of the presence of epilepsy.

A structured questionnaire, including many demographic and disease-related factors, was administered to all the study participants. The sociodemographic factors included marital status, employment status, nationality, socioeconomic level (poor, medium, or high), and educational level. Patients were asked to which socioeconomic level they belong. Regarding the educational level, participants were asked about the last degree they received; those who weren’t able to read and write were considered illiterate, those who were current school students or school graduates were considered school-level, and those who were university students or university graduates were considered university-level. Disease-related factors included frequency of seizures (number of seizures in the last four weeks), type of seizures (focal, focal with secondary generalization, generalized, or undetermined), duration of epilepsy (new onset epilepsy versus more than one year), number of antiepileptic drugs (AEDs) (monotherapy; one AED, versus polytherapy; more than one AED), and presence of comorbidities other than epilepsy.

Data analysis

IBM SPSS Statistics for Windows, Version 23.0 (Released 2015; IBM Corp., Armonk, New York, United States) was used for data analysis. To check if there is a significant correlation between the QOL scores and numeric demographics (age), the Pearson correlation was used. To check if there is a significant difference between the QOL scores and the qualitative (non-numeric) demographics, an independent t-test was performed after transforming the qualitative parameters to numeric, i.e the married and unmarried groups were recoded as 0 and 1 respectively, and this method was used for all other qualitative parameters. The difference in the QOL between the groups is considered to be significant when the p-value is less than 0.05. The independent t-test was performed if the comparison was between two groups. If the comparison was between more than two groups, one-way ANOVA was performed. For instance, independent sample t-test and one-way ANOVA were used to check if there is any significant difference in the mean of the total QOL score between groups with different sociodemographic and clinical characteristics.

## Results

Our sample included 47 patients; the majority were males (53.2%), with a mean age of 37.54 ($\pm$14.55) years. Out of these patients, 38 (80.9%) were Lebanese and nine (19.1%) were non-Lebanese but residing in Lebanon, and they were mostly married (59.6%). According to the educational level, 34 (72.3%) were school-level, 12 (25.5%) were university-level, and only one patient (2.1%) was illiterate. As for employment status, 20 (42.6%) patients were employed and 27 (57.4%) were unemployed. In addition, the socioeconomic level of the patients was assessed and our sample belonged mostly to the medium socioeconomic level, where 32 participants (68.1%) had a medium income per month and 15 (31.9%) had a poor income. It is to be noted that none of the patients belonged to the high socioeconomic level. Table 1 and the "education level" section of Table 2 show the sociodemographic characteristics of our sample. 

**Table 1:** Comparison in the means of total QOL scores among subgroups of different sociodemographic characteristics p-value less than 0.05 is considered significant; ˚independent sample t-test QOL: quality of life

	Sociodemographic characteristics	N(%)	Total QOL score mean (SD)	95% Confidence Interval	p-value
Gender	Male	25(53.2)	65.56 (22.31)	(-9.60, 13.31)	0.746˚
	Female	22(46.8)	63.70(15.57)		
Marital status	Married	29(59.6)	64.77(18.87)	(-11.48, 11.85)	0.975˚
	Single	19(40.4)	64.58(20.37)		
Employment	Employed	20(42.6)	74.70(11.38)	(7.89, 26.96)	0.001˚
	Unemployed	27(57.4)	57.27(20.69)		
Socioeconomic level	Poor	15(31.9)	55.34(22.8)	(-25.29, -2.16)	0.021˚
	Medium	32(68.1)	69.07(16)		
Nationality	Lebanese	38(80.9)	68.87(16.16)	(8.83, 34.80)	0.001˚
	Non-Lebanese	9(19.1)	47.05(22.21)		

**Table 2:** ANOVA results for the total QOL score among groups of the education level and type of seizure p-value less than 0.05 is considered significant; ®one-way ANOVA QOL: quality of life

	ANOVA subgroups	N (%)	Total QOL score mean (SD)	95% confidence interval for mean (lower bound, upper bound)	F-value	P-value
Education level	illiterate	1 (2.1)	38.44 (-)	-	0.95	0.395®
	School	34 (72.3)	65.11 (19.94)	(58.18,72.07)		
	University	12 (25.5)	65.68 (17.2)	(54.75,76.61)		
Type of seizures	Focal	9 (19.1)	68.94 (15.64)	(56.92, 80.96)	0.388	0.762®
	Generalized	27 (57.4)	63.86 (21.13)	(55.51, 72.22)		
	Focal with secondary generalization	5 (10.6)	58.05 (23.45)	(28.94, 87.17)		
	Undetermined	6 (12.8)	67.55 (13.18)	(53.72, 81.38)		

With respect to the clinical characteristics of epilepsy, the type of seizures differed among participants; most of the patients had generalized seizures (57.4%), nine (19.1%) had focal seizures, five (10.6%) had focal seizures with secondary generalization, and six patients (12.8%) had undetermined seizure types. Although a small group of patients reported comorbidities associated with their epilepsy (34%), most of our sample had no comorbidities (66%). Regarding antiepileptic drug treatment, most patients were on monotherapy and only 25.5% were on polytherapy. Most of our sample had controlled epilepsy, as 36(78.3%) patients had no seizures in the preceding four weeks. Furthermore, the majority of our patients (83%) had chronic epilepsy. Table 3 and the "type of seizures" of table 2 show the clinical characteristics of the patients. Concerning the clinical characteristics, our results showed no statistically significant association between any of the epilepsy-related factors (type and frequency of seizures, number of AEDs, duration of epilepsy, and presence of comorbidities), and the total QOL scores (Table 3).

**Table 3:** Comparison of the means of total QOL scores among subgroups of different clinical characteristics p-value less than 0.05 is considered significant; ˚independent sample t-test QOL: quality of life; AED: antiepileptic drug

	Clinical characteristics	N (%)	Total QOL score mean (SD)	95% Confidence Interval	p-value
Comorbidities	Yes	16 (34)	65.05 (16.4)	(-12.62, 11.53)	0.928˚
	No	31 (66)	64.5 (20.85)		
AED	Mono	35 (74.5)	65.3 (20.09)	(-10.72, 15.49)	0.715˚
	Poly	12 (25.5)	62.91 (17.33)		
Frequency	0	36 (78.3)	66.71 (17.86)	(-4.62, 21.92)	0.196˚
	At least 1	10 (21.7)	58.06 (22.98)		
Years since onset	More than 12 months	39 (83)	64.28 (20.27)	(-17.64, 12.79)	0.750˚
	Less than 12 months	8 (17)	66.70 (14.29)		

Results for the sociodemographic characteristics showed that there was a significant difference in the total QOL scores between those who are employed compared to those who are not (p-value =0.001), where the QOL is better in the employed patients. Similarly, there is a significant difference in the total QOL scores between patients of medium socioeconomic level and those with poor socioeconomic level (p-value =0.021), where the QOL of the medium group was higher than that of the poor group, and between the Lebanese and non-Lebanese nationalities where the Lebanese participants had a higher QOL total score compared to the non-Lebanese (p-value=0.001) (Table 1). However, age had no associations with the total QOL (data not shown).

Table 4 shows the correlation between the QOL subscales and different sociodemographic variables, where significant associations were detected between employment status and the following subscales; seizure worry, overall QOL, energy, medication effects, and social subscale (p-values<0.05). Significant associations were also detected between socioeconomic level and the overall QOL, emotional and social subscales. Similarly, nationality was significantly correlated with seizure worry, overall QOL, energy, medication effects, and social subscales. Further, the educational level was correlated with the social subscale (p-value =0.033) as shown in Table 4 while age was correlated with the seizure worry subscale (p-value=0.045) with a Pearson correlation of 0.296 (data not shown), where older patients tend to have less seizure worry.

**Table 4:** QOL subscales scores in sociodemographic subgroup p-value less than 0.05 is considered to be significant QOL: quality of life

	Sociodemographic groups	Seizure worry mean (SD)	Overall QOL mean (SD)	Emotional mean (SD)	Energy mean (SD)	Cognitive mean (SD)	Medication effects mean(SD)	Social mean(SD)
Gender	Male	59.27 (30.4)	65.8 (22.28)	59.68 (25.38)	63.2 (25.2)	64.35 (28.86)	70.05 (33.28)	71.14 (26.99)
	Female	51.88 (22.97)	65.23 (17.61)	57.45 (24.77)	54.55 (19.02)	66.6 (23.59)	51.7 (31.22)	74.88 (25.2)
	p-value	0.357	0.923	0.763	0.195	0.773	0.058	0.924
Marital status	Married	51.17 (26.44)	67.32 (18.72)	57.43 (25.42)	57.5 (22.13)	65.87 (23.69)	61.7 (32.41)	75.12 (25.99)
	Single	56.75 (28.86)	62.89 (22.04)	60.42 (24.55)	61.58 (23.92)	64.71 (30.34)	61.11 (35.45)	73.56 (26.42)
	p-value	0.846	0.463	0.69	0.551	0.884	0.953	0.842
Employment	Employed	70.15 (19.99)	74.38 (17.92)	65 (20.8)	70.75 (16.33)	71.64 (20.31)	74.72 (29.89)	89.65 (12.7)
	Unemployed	45.18 (27.13)	58.98 (19.22)	53.93 (26.86)	50.56 (23.18)	60.79 (29.45)	51.64 (32.76)	63.25 (27.56)
	p-value	0.001	0.008	0.118	0.002	0.142	0.017	0.000086
Socioeconomic level	Poor	46.67 (32.37)	57 (22.26)	46.67 (23.45)	54.67 (27.78)	59.77 (33.06)	54.26 (31.21)	58.59 (26.66)
	Medium	60.09 (23.68)	69.53 (17.85)	64.25 (23.78)	61.25 (20.12)	68.05 (22.52)	64.84 (34.18)	81.94 (22.22)
	p-value	0.115	0.044	0.022	0.36	0.39	0.315	0.003
Nationality	Lebanese	63.8 (22.65)	69.87 (18.17)	60.63 (23.48)	64.87 (18.21)	68.26 (24.75)	66.96 (30.64)	79.29 (23.4)
	Non-Lebanese	22.07 (16.75)	47.22 (17.61)	50.22 (30.01)	35 (24.87)	53.35 (30.52)	38.27 (35.86)	54.22 (27.39)
	p-value	0.000005	0.002	0.263	0.000161	0.127	0.018	0.008
Educational level	Illiterate	34.66 (-)	40 (-)	44 (-)	45 (-)	38.66 (-)	44.43 (-)	30 (-)
	School	54.71 (26.71)	66.91 (20.02)	59.29 (24.27)	60.74 (22.8)	68.09 (26.18)	61.76 (29.91)	71.17 (27.04)
	University	60.7 (29.5)	63.75 (20.1)	58 (28.16)	55.83 (23.63)	60.02 (26.64)	62.04 (43.88)	87.58 (14.44)
	p-value	0.601	0.398	0.834	0.676	0.396	0.879	0.033

Furthermore, no significant correlation was detected between any of the clinical characteristics of epilepsy and various QOL subscale scores (Table 5). 

**Table 5:** Correlation between QOL subscales scores and clinical characteristics of patients p-value less than 0.05 is considered to be significant QOL: quality of life; AED: antiepileptic drug

		Seizure worry mean (SD)	Overall QOL mean (SD)	Emotional mean (SD)	Energy mean (SD)	Cognitive mean (SD)	Medication effects mean (SD)	Social mean (SD)
Type of seizure	Focal	65.63 (28.38)	7.83 (17.81)	65.33 (27.93)	51.67 (25)	71.76 (17.63)	61.73 (39.67)	78.81 (16.31)
	Generalized	52.2 (27.82)	65.74 (21.5)	55.85 (24.97)	62.41 (22.68)	59.99 (28.52)	63.32 (32.68)	77.05 (29.98)
	Primarily focal with secondarily generalized	41.33 (21.28)	64.5 (20.8)	52 (19.18)	52 (26.6)	67.89 (32.51)	42.78 (28.71)	57.45 (29.27)
	Undetermined	51.39 (26.36)	57.5 (17.25)	66.67 (16.93)	61.67 (16.93)	78.15 (18.48)	68.28 (32.33)	70.67 (17.64)
	p-value	0.441	0.67	0.591	0.565	0.382	0.605	0.433
Comorbidities	No	54.1 (30.15)	64.11 (19.01)	60 (23.55)	59.03 (26.56)	64.13 (27.01)	57.79 (35.96)	76.43 (27.66)
	Yes	59.12 (20.57)	68.28 (22.21)	56 (27.79)	59.38 (13.02)	67.88 (25.42)	68.57 (27.04)	70.71 (22.39)
	p-value	0.553	0.505	0.606	0.953	0.647	0.298	0.478
AED	Mono	54.1 (28.88)	66.57 (19.6)	60.46 (24.9)	58.71 (24.23)	65.54 (27.24)	60 (36.37)	76.98 (26.77)
	Poly	60.81 (27.58)	65.42 (22.02)	53.33 (24.97)	60.42 (18.4)	65 (24.3)	65.74 (22.83)	67.21 (22.58)
	p-value	0.466	0.982	0.397	0.825	0.952	0.529	0.264
Years since onset	More than 12 months	58.7 (27.1)	65.13 (20.6)	58.1 (26.16)	59.5 (21.6)	62.9 (26.55)	63.19 (33.63)	75.28 (25.93)
	Less than 12 months	39.2 (22.33)	67.86 (17.4)	61.71 (16.47)	57.14 (30.26)	79.6 (20.72)	51.59 (31.87)	69.93 (27.17)
	p-value	0.080	0.743	0.727	0.803	0.122	0.401	0.619

## Discussion

The aim of our study was to assess the QOL of epilepsy patients in Lebanon, and the relation of the sociodemographic and clinical characteristics to the QOL and its subscales. Our results showed that the highest subscale score was for social functioning and the lowest was for the seizure worry subscale, reflecting the negative impact of seizure worry on the QOL of epileptic patients. In Malaysia and Africa, the highest scores were for medication effects and the lowest scores were also for seizure worry, similar to ours [7,18]. The mean total score for our sample was 64.6885±19.27858, only slightly lower than the study conducted in Malaysia (68.9±15.9), which could be due to the higher standards for medical care in the latter [18], but higher than that of Benin (52.1±33.4) and Togo (49.5±14.4) in Africa [7], similarly reflecting the difference in standards of care between countries. The mean QOL total score was also higher than that of a study performed in Iraq (47.9±18.1), which used a different questionnaire (SF-36); the difference in scores may be due to the fact that they used a different questionnaire or due to the generally controlled symptoms in our sample [15]. Epileptic patients in our study were more likely to be unemployed, married, and of medium economic level. In terms of employment status, it resembles the Iraqi study [19], where their patients were also more likely to be unemployed, and this was further emphasized by other studies in which epileptic patients tend to be underemployed [20]. However, unlike other studies [7,19,21], there was a higher percentage of married participants in our study; this may be due to the fact that our sample has a median age of 37.54 and are generally more likely to be married.

Only 10 patients (21.7%) in the current study experienced at least one seizure in the past four weeks, which is lower than the case in Malaysia where almost half of their patients experienced seizures [18], and also lower than that in the study conducted in Iran, the Gulf, and Near East where 42% experienced seizures [22]. This probably reflects the selection of patients who were well controlled in our sample and taken from general neurology clinics, unlike the study done in Malaysia, where patients were enrolled from a tertiary care center, where certainly more refractory patients were seeking care.

With respect to the total QOL scores, significant differences were observed between the employed and non-employed participants, poor and medium socioeconomic-level participants, and Lebanese and non-Lebanese participants as well, where better QOL total score was observed among employed, Lebanese, and medium socioeconomic-level participants. This is unlike the findings in the Malaysian study, where none of the sociodemographic characteristics was correlated to the QOL score [18], but is supported by several other studies. For instance, the study performed in Iran, the Gulf, and Near East, has included Lebanon and has shown that the QOL of epileptic patients in Lebanon was generally good [22]. This can help emphasize either that patients in Lebanon were well controlled or that being Lebanese was associated with a better QOL. A recent study performed in Lebanon also reported that employment improved the QOL of people suffering from epilepsy [4], emphasizing our results, and another study conducted in Iraq showed that socioeconomic level was strongly correlated with the QOL [19]. This highlights the importance of financial satisfaction and wellbeing in the QOL of epilepsy patients in Lebanon. Furthermore, being employed and having a medium socioeconomic level not only gives the participant a sense of productivity and stability but also provides the means necessary to get medical support, in terms of being able to visit the neurologist and to buy the AEDs needed for seizure control.

On the other hand, there was no statistically significant difference between males and females, similar to the study performed in Northwest Greece [6], but as opposed to the studies conducted in Turkey and Georgia, where females had poorer QOL compared to males [21,23]. No difference was similarly observed between married and single patients; this either signifies that support does not play a crucial role in the QOL of patients or that single and married groups both are supported or between educational level groups, unlike studies in Georgia and Iraq where lower educational level predicted poorer QOL [19,21]. Age seemed to have no effect on the total QOL score as well, which is supported by some studies [6,23], but is contradictory to an Australian study and another study performed in Lebanon, where advanced age was associated with a poor QOL [4,24]. This has to do with the fact that our sample is generally young. 

Surprisingly, there was no relationship between any of the epilepsy-related clinical features and the QOL total score. This was in contrast to most other studies, where seizure frequency was found to be a predictor of the HRQOL of epileptic patients [7,18,19,21-23,25,26]. This could be related to the selection of a relatively well-controlled epilepsy group in our cohort, but also to our small sample size. Besides, our result was similar to that of a study conducted in Iraq, which showed that the number of AEDs didn’t affect the QOL scores, although the QOL of participants on monotherapy was slightly higher than those on polytherapy [19]. However, this was opposing several other studies showing that polytherapy was associated with more side effects and affected negatively the QOL of patients [4,8,22,27].

In our sample, age was correlated with the seizure worry subscale. This correlation although weak but indicates that as patients suffering from epilepsy get older, their worry about seizures decreases. In other words, younger age may be associated with more concern about having a seizure unpredictably, knowing that the younger population is more likely to be involved in activities that might actually put them at risks, such as driving, swimming, diving, and flying activities. Moreover, it often takes time for them to adapt to the fact that they have a chronic neurological condition that requires caution and abstinence from certain activities. This is in contrast to the Georgian study, where age was shown to be negatively correlated with the overall QOL score [21].

In addition, the socioeconomic level was shown to be correlated to the overall QOL, emotional wellbeing, and social functioning subscales. Moreover, educational level was shown to be correlated to the social subscale, which is reflected by the rise of the scores in relation to the educational level where the school-level participants showed lower scores than the university-level participants. Similar results were shown in studies by Siarava et al. and Djibuti et al. [6,21].

None of the clinical characteristics of epilepsy seemed to be associated with any of the QOL subscale scores, in contrast to several studies that had shown that a higher seizure frequency [4,18,22,25,26], presence of comorbidities [4], or types of seizures [26] correlated with a poorer QOL [28]. This could be mainly due to the small sample size, but also could have been related to the selection of our cohort, where the majority of our patients were on monotherapy, seizure-free, had generalized seizures, and had no comorbid conditions.

Finally, several studies reported that QOL scores increase with time due to the adaptation factor [21,25,26]; however, this could not be proven in our study, since there was no significant association detected between the 'years since onset' variable and any of QOL subscale scores. This could be because of the small number of patients in general and also the smaller number of patients belonging to the new onset group (17%), in comparison to the chronic group (83%). Similar results were reported in studies done in Turkey and Malaysia [18,23], where also there was an absence of a significant difference in the QOL scores between the new-onset and chronic subgroups.

Limitations 

The most important limitation of our study is the small sample size (47), which prevented the detection of significant correlations between different variables, mainly epilepsy-related factors, with QOL total scores and subscales. Another important limitation is the fact that the sample is not representative of the whole Lebanese population, since most of our patients were taken from two governorates (Beirut and Mount Lebanon). The other crucial limiting factor was our use of the older classification for the types of seizure (not ILAE updated 2017 classification) since the participants contacted were already diagnosed by the previous classification and it was hard for us to update their files at the time of enrollment. In addition, the QOLIE 31 questionnaire used in our study is a subjective one filled by participants, who may be hesitant to share personal information, and the socioeconomic level was determined through questions asked to the patient, not based on a certain income level. Hence, responses might be highly subject to reporting bias and may not be very precise and transparent.

## Conclusions

Our results showed that sociodemographic factors played a major role in determining the QOL of epileptic patients in Lebanon. For instance, being Lebanese, employed, and belonging to the medium socioeconomic level were associated with better QOL scores. This highlights the importance of financial and social stability in determining the QOL of patients in our sample. However, further studies on a larger sample size may be required to emphasize our results. Furthermore, QOL assessment must be included in the long-term management of all patients suffering from this chronic disease, and measures must be implemented to improve their QOL.
